# Supplementary material for: Comprehensive Annotation and Functional Exploration of MicroRNAs in Lettuce
Source: Front Plant Sci. 2021 Dec 24;12:781836. doi: 10.3389/fpls.2021.781836 (PMC8739914; doi:10.3389/fpls.2021.781836)
Supplement: Supplementary file 3 [file Data_Sheet_9.ZIP › GSM2230753.plot/Lsa-miR159b_Lsat_1_v5_gn_8_18560.1_254_TPlot.pdf]

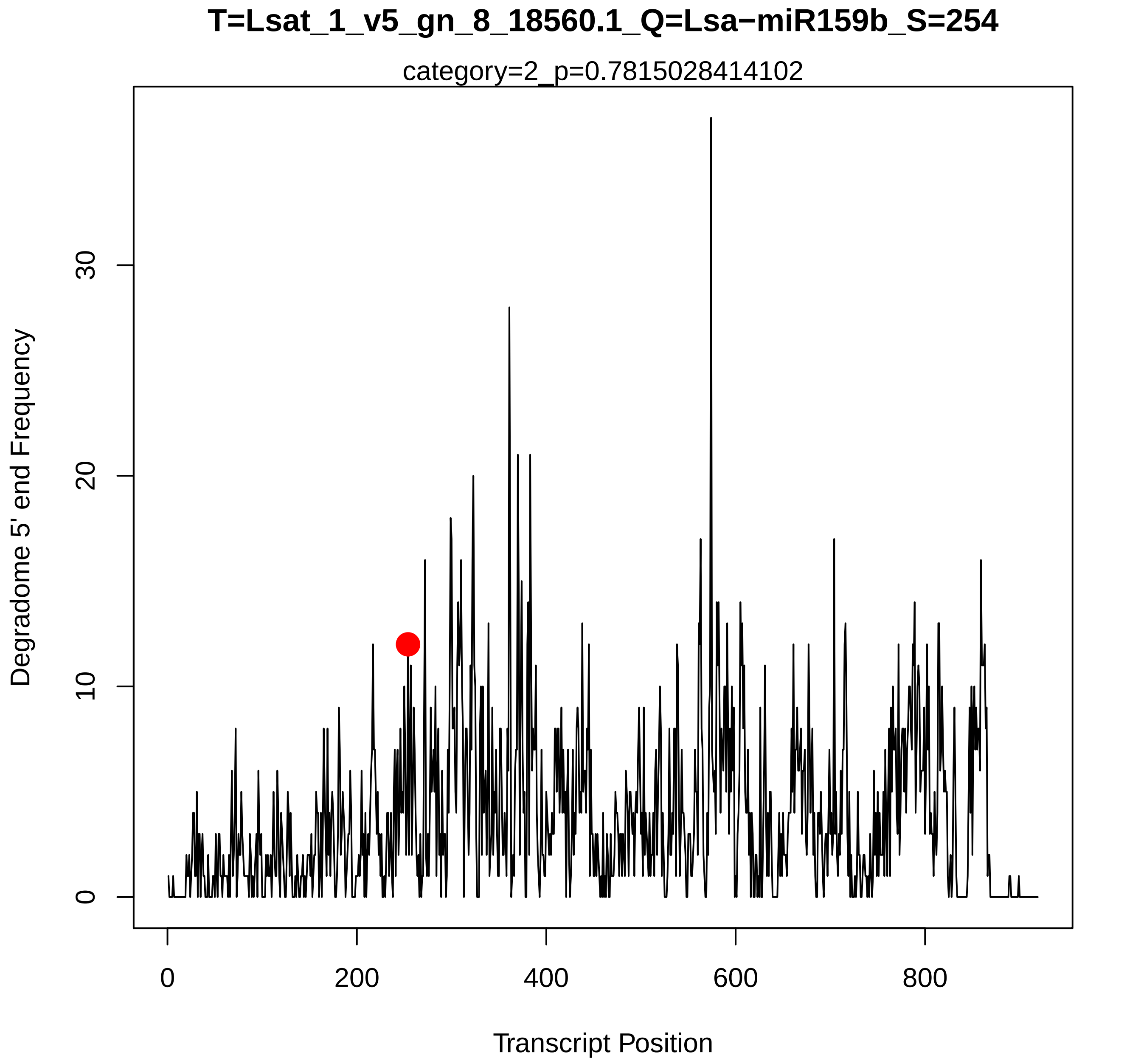
T=Lsat_1_v5_gn_8_18560.1_Q=Lsa−miR159b_S=254
category=2_p=0.7815028414102
Degradome 5' end Frequency
0
10
20
30
0
200
400
600
800
Transcript Position
